# Housing as a Determinant of Tongan Children’s Health: Innovative Methodology Using Wearable Cameras

**DOI:** 10.3390/ijerph14101170

**Published:** 2017-10-04

**Authors:** Andrew Robinson, Sarah Hulme-Moir, Viliami Puloka, Moira Smith, James Stanley, Louise Signal

**Affiliations:** Health Promotion & Policy Research Unit, University of Otago, Wellington 6242, New Zealand; roban653@student.otago.ac.nz (A.R.); hulsa010@student.otago.ac.nz (S.H-M.); Viliami.puloka@otago.ac.nz (V.P.); Moira.smith@otago.ac.nz (M.S.); James.Stanley@otago.ac.nz (J.S.)

**Keywords:** Tonga, housing, children, health impact

## Abstract

Housing is a significant determinant of health, particularly in developing countries such as Tonga. Currently, very little is known about the quality of the housing in Tonga, as is the case with many developing countries, nor about the interaction between children and the home environment. This study aimed to identify the nature and extent of health risk factors and behaviours in Tongan houses from a child’s perspective. An innovative methodology was used, Kids’Cam Tonga. Seventy-two Class 6 children (10 to 13-year-olds) were randomly selected from 12 randomly selected schools in Tongatapu, the main island. Each participating child wore a wearable camera on lanyards around their neck. The device automatically took wide-angled, 136° images of the child’s perspective every seven seconds. The children were instructed to wear the camera all day from Friday morning to Sunday evening, inclusive. The analysis showed that the majority of Tongan children in the study live in houses that have structural deficiencies and hazards, including water damage (42%), mould (36%), and electrical (89%) and burn risk factors (28%). The findings suggest that improvements to the housing stock may reduce the associated health burden and increase buildings’ resilience to natural hazards. A collaborative approach between communities, community leaders, government and non-governmental organisations (NGOs) is urgently needed. This research methodology may be of value to other developing countries.

## 1. Introduction

The Kingdom of Tonga is a Pacific Island state with an estimated population of 103,000 (2017), of which 39% are aged 15 years or younger, while only 8% are 60 years and older [1]. Housing is a significant determinant of physical, mental, and social wellbeing among children [2,3]. Across developing countries, children in low-income families are more likely to live in housing which exposes them to a multitude of health risks associated with the development of infection, respiratory illness, cardiovascular disease, and injury [2,4].

Pacific Islands have the additional burden of improving housing and infrastructure in the face of unpredictable severe weather patterns and rising sea-levels [5]. The Pacific Climate Change Science Program estimates that the wind speeds of Pacific cyclones are set to increase 11% this century and rainfall intensity will increase 20% in the same period [5]. Cyclones have the most frequent and damaging impacts on housing, and result in the deterioration of the health of affected populations [6]. The direct harms are caused by injury and trauma from collapsing houses and flying debris [6]. This is of particular concern in Tonga, where the construction of Western-style housing has gradually usurped the traditional Tongan *fale* (house), which is both more storm resistant and more easily rebuilt [7]. The last major cyclone (cyclone “Ian”) in 2014 rendered 2300 people homeless (1130 buildings) [8]. It was widely reported that Western-style houses sustained an overwhelming proportion of damage, with an estimated cost of U.S. $48 million, while the traditional Tongan *fale* remained unaffected [9].

With increased rainfall, there is also an increase in mosquitos and pests, which breed in stagnant water and unhygienic environments [5,10]. Tonga has had previous outbreaks of dengue fever, chikungunya virus (over 10,000 cases), and Zika virus (unconfirmed) [11]. Households can protect against the transmission of vector-borne disease by having mosquito nets over sleeping areas, window screens on homes, and appropriate water management and drainage [12]. Aside from vector-borne diseases, Tonga has seen dramatic reductions in communicable diseases and child mortality in the last 50 years [13]. This is thought to be in part due to improved hygiene in the home environment; specifically, increased access to potable drinking water and waste removal and sewage treatment systems [13]. Despite recent improvements to households and amenities, respiratory illness (not related to smoking) remains the third most common admission to hospital, followed by infectious and parasitic diseases, which includes the vector-borne diseases [14].

Multiple international studies have shown a significant relationship between visible mould, dampness, and respiratory illness, specifically asthma [2,3]. Tropical regions with high humidity and damp housing provide the optimal environment for mould growth [15], and asthma is now the eighth leading cause of disability in Tonga [16]. Moulds associated with damp homes are often allergenic and are recognised as a leading cause of childhood asthma [3,17]. In addition to asthma, acute rheumatic fever (ARF) remains a significant problem in Tonga, with the incidence reported as 13.4 per 100,000 (The Organisation for Economic Co-operation and Development (OECD) average 1.2 per 100,000) and prevalence of rheumatic heart disease (RHD) 33.2–42.6 per 1000 [18,19]. Whilst the etiology and causes of rheumatic fever are not well understood, it has been strongly linked to overcrowding and poorer housing condition [20]. In Tonga, the average household size is 5.7 persons per household, compared to the OECD average of 2.6 [21]. Whether this constitutes overcrowding would depend on the amount of household space and how that space is used [22]. There is currently very limited research in the area of housing in Tonga, and very little is known about the quality of the housing or the interaction between children and the home environment.

Typically, housing has been studied by third party observation [23]. Kids’Cam is a methodology to assess the world in which children live and their interaction with it [24], providing researchers with the opportunity to study children’s environment from their perspective [24]. This design makes it possible to identify the influences and behaviours in the children’s world, which may have positive or negative impacts on the children‘s health and wellbeing [24]. The Kids’Cam methodology has previously been used with New Zealand children to explore the marketing of unhealthy foods and a range of other public health issues. These include studies of children’s exposure to food marketing [25], “blue” space [26], smoking, and alcohol, as well as the use of “green” space, transport, and sun protection. Kids’Cam Tonga is a parallel study based on Kids’Cam methodology [24] that aims to study the world in which Tongan children live and their interaction with it. The research reported here used the Kids’Cam Tonga data from the main island of Tongatapu to identify the nature and extent of health risk factors and behaviours in Tongan houses from a child’s perspective.

## 2. Methods

### 2.1. Study Design

Kids’Cam Tonga is a cross-sectional observational study. In the Kids’Cam Tonga Tongatapu study, seventy-two Class 6 children (10 to 13-year-olds) wore a wearable camera (Autographer, U.S. $280) on lanyards around their neck [24]. The device automatically took wide-angled, 136° images of the child’s perspective every seven seconds. Full details of the original Kids’Cam study methods and the study protocol are published elsewhere [24].

Kids’Cam Tonga is a collaboration with the Tongan Ministry of Education and Training, the Ministry of Health, and the Tonga Health Promotion Foundation, TongaHealth. Support for the study, and advice on how best to conduct it, was sought from senior staff in these agencies by the Tongan research lead, Dr Viliami Puloka. The Ministry of Education and Training and TongaHealth assisted with the research as outlined below.

### 2.2. Ethical Approval

Ethical approval was obtained from the University of Otago Human Ethics Committee (Health) (13/220) and the Tonga National Health Ethics and Research Committee (#290116) to study any aspect of the world children live in and their interaction with it. All precautions were taken to ensure that participating children were protected from harm and the anonymity of participants and third parties respected, including in any published images where faces and identifying features are obscured. Further, published images of houses were carefully chosen, and then cropped or obscured, to ensure focus on the feature of interest and avoid unnecessary identification of the particular dwelling.

### 2.3. Sampling and Recruitment

A list of all schools (n = 55) in Tongatapu that included a Class 6 roll was obtained from the Tongan Ministry of Education and Training. Sampling was conducted in two stages. First, twelve schools were randomly selected by location on the basis of probability-proportional-to-size sampling methods, stratified by region: six urban schools (Nuku’alofa) and six rural schools (Eastern and Western Districts). Next, children were randomly selected from each of these schools. Sample size was determined prior to the study based on the total number of schools and children that could be included within the project budget.

Officials from the Tongan Ministry of Education and Training facilitated school recruitment. School principals and/or senior teachers were contacted to explain the study and its purpose, and the data collection procedure. Written consent was gained from the principal of each school. The list of randomly selected children was given to the school principal, who then approached the selected children and their families, inviting them to participate and providing them with information sheets and consent forms. The six children and their parents at each school who provided written consent to participate attended a briefing session on the day prior to the start of data collection to explain the project. Children and parents were advised in writing, and in person, that their images might be used in subsequent publications, but that every effort would be made to protect their anonymity.

### 2.4. Data Collection and Management

The children were instructed to wear the camera all day from Friday morning to Sunday evening, inclusive. This timeframe was selected to capture the children's environment on a school day, and their home, church, and recreational environments. Following data collection, cameras were collected and images downloaded, with children given the opportunity to review and delete any photos they did not want the researchers to see. This was done to ensure privacy. If in fact the children did delete any images, they appeared to delete very few. Children were compensated for their time with book vouchers and given certificates of participation. Schools were also compensated with book vouchers. Approved images were downloaded to a password-protected server, saved in secure cloud storage, and backed up to a password-protected external hard drive. Data access was restricted to members of the research team who had agreed to adhere to strict protocols regarding the release and use of data.

### 2.5. Coding of Image Data for Housing Study

The images were viewed on a standard 22-inch PC monitor, with two images side-by-side on the screen at a time (image size on screen of 12 cm by 9.5 cm). Two trained investigators independently identified and coded all of the images that contained a participant's house and surrounding environment. In cases where the participant's house was not immediately obvious, care was taken to identify the place where they spent the most time undertaking activities (sleeping, washing clothes, eating evening meal), suggestive of a permanent home environment. The house was assessed qualitatively for specific factors under the following categories: building materials, structural quality, risk factors and behaviours, and amenities and functions. Key terms and definitions can be found in the coding schedule (Table S1). As there is no standardised tool for assessing housing use in the Pacific Islands, a New Zealand tool was adapted by the research team for Tongan conditions [23]. Factors were only coded once for a given house, with the positive identification of a specific factor recorded as “1” in the coding schedule and no identification (or evidence) of a specific factor recorded as “0”. Reliability testing was conducted between the two independent, trained investigators, and achieved an 82% concurrence when all the images in the home environment of one child were assessed and the results compared.

### 2.6. Key Stakeholder Workshop and Finalising of Results

Draft findings were presented at a workshop in Nuku’alofa by Puloka and Signal. The meeting was organised by TongaHealth and attended by 15 key stakeholders. Senior staff from key ministries (including the Ministry of Infrastructure), TongaHealth, and non-governmental organisations (NGOs) attended. Feedback was sought on this analysis and related Kids’Cam Tonga research on non-communicable disease risk factors. Following the incorporation of feedback, a draft of this paper was sent to key advisors for input and final approval.

## 3. Results

The Kids’Cam methodology was effective at capturing the nature and extent of health risks and behaviours in the home environment from the children’s perspective. The images provided descriptive evidence of the quality of the housing, structural damage, water damage, the cooking environment, electrical hazards, sleeping arrangements, and additional functions of the home.

All 72 children invited to participate did so, and there was no drop-out. The children collected approximately 700,000 images. The photo review took participants an average of 40 min, and photos were rarely deleted. The trained investigators took 200 h to review all of the photos for housing and analyse the housing images in detail. The vast majority (95%) of the housing images could be coded. Where this was not possible, sometimes images taken at other times enabled coding. By coding all of the images collectively for any given child, a reasonably comprehensive perspective of the risk factors and behaviours could be ascertained. Of the 72 houses included in the study, 40 (56%) were located in a rural region of Tongatapu and 32 (44%) were located in an urban region (Nuku’alofa). In 2011, 31% of the population in Tongatapu were recorded as living in Nuku’alofa, Tonga’s only urban region [21].

Key stakeholders welcomed these findings, noting that most of the information was not currently known. The key changes requested were: (1) to ensure that a balanced picture of housing was presented, i.e., that many houses in Tonga are structurally sound; and (2) that any published images were anonymized. Stakeholders requested that findings be made available to the community in order that action could be taken. They congratulated the research team on their collaborative approach, particularly on taking the time to present the findings in Tonga. Overall, they noted the value of the image data, including its ability to add meaning to statistical data already available.

### 3.1. Description of Housing

Houses were constructed from non-traditional, imported building materials: cinder blocks, plasterboard, lumber, chipboard, and corrugated metal sheet. Of the 72 houses coded, none were traditional Tongan *fale* and very few houses used the locally sourced materials of coconut fibre, *kamani* (*Gluttiferae*)*, kou* (*Cordia subcordata*), and *vesi* wood (*Intsia bijuga*). The exterior walls and cladding were made of cinder block or concrete in 33 houses (46%), lumber in 33 houses (46%), and corrugated metal sheet in the remaining six houses (8%). The predominant roofing material was corrugated metal sheet, used in 71/72 houses (99%). The houses were constructed in a square shape, with usually one point of entry at the front of the house, and occasionally a second point of entry at the rear of the house. A number of houses had building materials (cinder blocks and metal sheet) piled up on the property, which suggested that they were undergoing structural renovations or improvements.

### 3.2. Structural Quality, Watertightness, and Mould

#### 3.2.1. Structural Damage and Water Tightness

**Figure 1:**
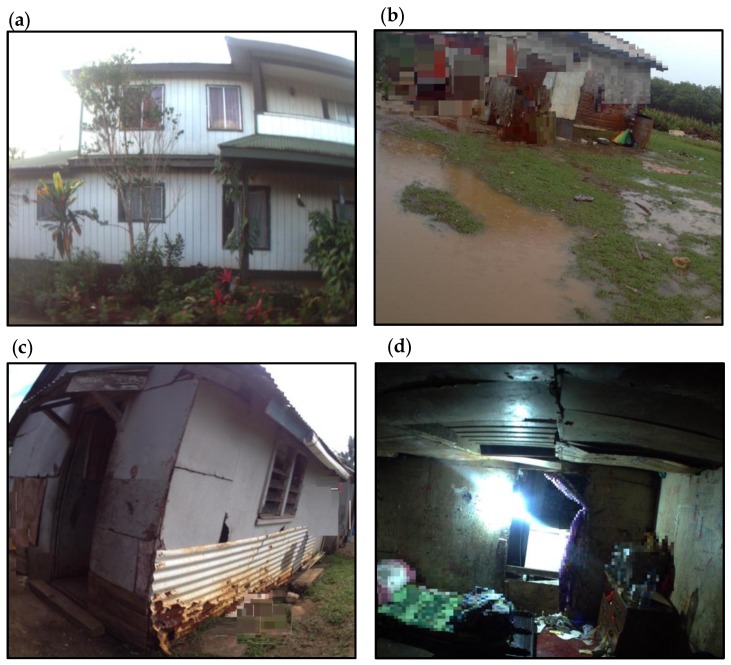
Range of house quality observed: (**a**) House without any evidence of structural damage and exterior is well maintained; (**b**) House surrounded by pools of rain water due to insufficient drainage, interior of house water damaged; (**c**) Poor building quality indicated by holes in the external wall and use of building materials to patch up exterior; (**d**) Children’s bedroom with damaged ceiling and walls, and evidence of water staining on the cardboard covering holes in the ceiling.

Over a quarter of the houses had no evidence of structural damage or water permeation into the home (Figure 1a). Eleven houses (15%) had significant and extensive structural damage, which could present a significant risk to the health of the occupants (Table 1). These houses did not appear structurally secure or weather tight, and would not provide adequate shelter from the outside environment (Figure 1b–d). Housing with poor structural integrity also incorporated low quality, informal materials to patch up internal walls and ceiling holes; examples included cardboard, tarpaulin, and plastic wrap. The most prevalent structural issue was the evidence of water damage in 42% of houses (Table 1). The water damage in these houses was indicated by rotten wood, water staining on a ceiling and walls, peeling paint, rusted metal, damaged flooring, and the use of plastic sheets or buckets to capture residual water flow. In six houses, the sleeping areas were in rooms with holes in the ceiling or walls, and there was insufficient natural lighting so the sleeping area appeared dark during daylight hours (Figure 1d). In 32% of the houses, the windows were permanently boarded up with wood or the windows were damaged so that they no longer provided a physical barrier to the outside environment (Table 1). The cause of the damage observed in many houses was suggestive of high-velocity wind damage, particularly for those houses with missing corrugated roofing and a loss of windows. Houses where the damage was less extensive were likely to be due to the progressive degradation of low-quality building materials.

#### 3.2.2. Cooking Sheds

**Figure 2:**
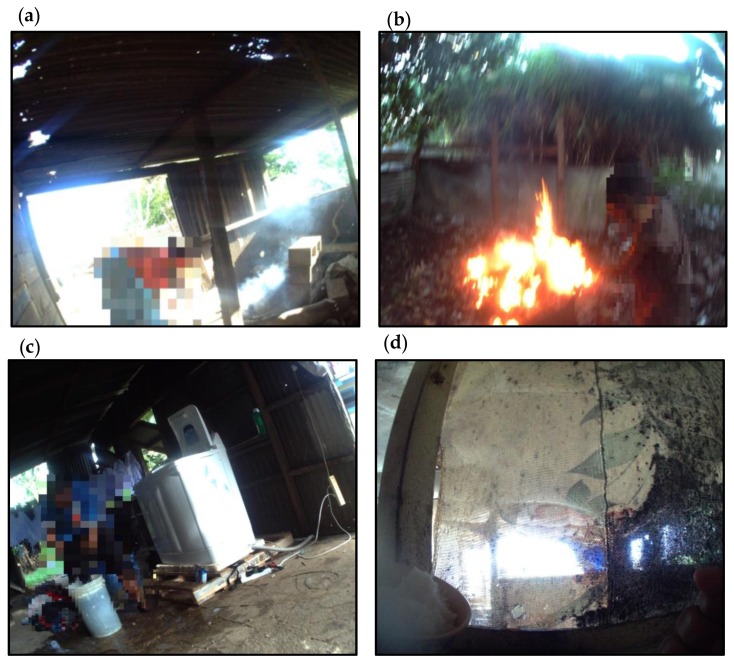
Risk factors and behaviours observed in the home environment that increase the risk of injury or harm to children: (**a**) Cooking on an open fire in a cooking shed filled with smoke. Low-quality building materials used in structure; (**b**) Child burning rubbish and standing near flame; (**c**) Electrical extension blocks and power cables next to a washing machine and in the vicinity of a water spill; (**d**) Fabric covered in mould next to a food preparation surface.

Nine houses did not have a formal kitchen, but had a separate cooking shed that was physically detached from the house (Table 1). The cooking sheds were more roughly constructed from rusted metal sheets and lumber. Seven of the nine cooking sheds identified had poor structural integrity, indicated by damaged walls and ceilings and evidence of water damage (Table 1). The sheds had limited storage and food preparation space, often contained large amounts of waste, and were easily accessible to dogs, pigs, or other animals kept around the home. Families spent a reasonably large proportion of their time in these cooking facilities throughout the day, sometimes spending more time in the cooking shed than the actual house. Families would prepare food in these sheds on the ground or in plastic buckets. If a gas cooker was not present, then some families cooked on an open solid fuel fire in the shed (Figure 2a). This was observed in 4% of households (Table 2).

#### 3.2.3. Mould and Ground Water Damage

Mould was visibly present in 36% of houses, predominantly in the cooking and sleeping areas (Table 1). In the cooking areas, mould was observed on the the walls associated with sinks, cookers, and windows. In the sleeping areas, mould could be observed on fabrics (curtains) and surrounding windows. The study participants would consume food, which had been prepared in areas of high mould density, and would touch walls and fabrics where mould was present (Figure 2d). The presence of mould usually coincided with evidence of water damage. In most images, it was not possible to assess active water infiltration due to the dry weather conditions at the time, but a few images showed water pooling around properties following rainfall (Figure 1b). Only 7% of houses were constructed on struts as a protective measure against ground water damage (Table 3). Rainfall was also important for household water use, with 56% of houses having a concrete water tank to store rainwater captured on the corrugated roofing (Table 3).

### 3.3. Risk Factors and Behaviours Observed in Households

#### 3.3.1. Electrical Hazards

All of the houses that were assessed had mains electricity and used electricity for lighting and general household appliances. For some homes, electrical circuits were built into the internal housing structure with electrical wall sockets and ceiling lighting. The main source of lighting was fluorescent bulbs, which were present in 89% of houses (Table 2). For the most part, extension cords were used to distribute power around the house. The informal layout of power leads from inside to outside of the house, along with electrical leads that were exposed and open to water, present a significant risk for electrical burns, fire ignition, and impact injury due to tripping over cables (Figure 2c). The overloading of multiplug sockets and relay of a multiplug-on-multiplug arrangement was common. The demands on multiplugs came from television sets, lighting, phone chargers, music stereos/speakers, washing machines, refrigeration, deep freezers, and other kitchen appliances.

#### 3.3.2. Cooking and Associated Risk Factors

Although electricity was widely used in the kitchen, only 13% of households had an electrical cooker (Table 3). Gas cookers were the predominant method of cooking, and these were used by 54% of homes (Table 3). Gas cookers require Liquified Petroleum Gas (LPG) cylinders for fuel, and these were visibly present inside of 21% of houses (Table 2). The majority of gas cylinders were located in the cooking areas, but some were also observed in the sleeping areas and living areas. A few families had used an inventive solution to safely store the gas cylinder outside by running the gas hose through the external wall to the cooker (Figure S1). In 7% of homes, informal gas cookers were observed, specifically portable camping stoves or barbeques. These systems are not designed for indoor cooking, as they leak harmful gases and present a fire risk. In three homes, indoor cooking was observed with solid fuels (wood and coconuts), and the images contained evidence of smoke buildup in designated, enclosed cooking areas (Figure 2a).

For the most part, cooking on solid fuel fires and barbeques was done outside of the built space. Children were actively involved in igniting fires and cooking food. In 28% of homes, there were behaviours that demonstrated an increased risk to burns or scalds (Table 2). Risks included: igniting highly flammable, toxic materials (petrol and plastics), children playing close to open fires unsupervised (Figure 2b), and active stove tops with metal cooking pots in hazardous positions. The risk of harm from household fire would be raised due to the reliance of flammable cooking fuels (gas), flammable building materials (lumber), and a lack of smoke alarms, as none were observed in any of the houses. Children were also actively involved in food preparation, but hand washing before the preparation or consumption of food was infrequently seen. Children would often prepare hard vegetables and meat using machete-style knives or large serrated knives. In 15% of homes, knives were seen left out on the ground or low-lying surfaces where they could be picked up by an infant (Table 2).

#### 3.3.3. Interaction with Home Environment and Associated Risk Factors

Children were also involved with household tasks such as cleaning, maintenance, water collection, and the washing of laundry. The maintenance of the property involved the gathering and burning of rubbish. At least 21% of households had waste on the floor of the house or dispersed around the property (Table 2). In these households, there appeared to be a lack of bins or recycling systems. Children were exposed to hazards which could cause physical injury in a number of households. In 14% of houses, there were large objects at heights above the level of the camera which appeared to be unstable or at risk of falling onto the child. These included fluorescent bulbs and light fittings hanging from the ceiling and television screens resting on top of stacked books. Broken glass was seen in 4% of houses, and scrap metal with sharp or rusting edges and exposed nails were identified in 22% of houses (Table 2). These hazards were usually in places where children were walking or at heights (above the level of the camera) that could cause injury.

The amount of time that children spent exposed to any given hazard or risk factor would be hard to estimate using the Kids’Cam images, as the image is restricted to the constraints of the camera view. Exposure to hazards may be lessened because the children spent most of their time outside of their house in the surrounding green space. When children were not at home, they were at school, church, other houses, or working at a parent's place of employment. The house was mainly used by children for socialising, watching television, eating meals, school work, and sleeping. In homes where it was possible to see the sleeping arrangement, there was a mixture of traditional sleeping and bedrooms with permanent beds. Traditional sleeping arrangements were seen in 40% of households, permanent bedrooms for children in 36% of households, and permanent bedrooms for adults in 31% of households (Table 3). Children who slept in a formal bedroom would share this space with other siblings, and the rooms usually contained a number of beds. The traditional sleeping arrangement consisted of temporary mattresses and sofas in the main socialising area. These were prepared in the evening and dismantled in the morning. A few adults appeared to be sleeping under mosquito nets, but overall mosquito nets were rarely seen. Further images are provided in Figures S1 and S2.

## 4. Discussion

The housing stock in Tonga has multiple structural hazards which may contribute to injury burden. One of the most alarming findings regarding the housing quality was the proportion of houses with significant structural deficiencies and water damage. The susceptibility of the housing to damage is attributed to the imported building materials and western-style design [6,27], with housing constructed as square builds with solid walls and corrugated metal roofing. When cyclone winds meet the resistance of vertical rigid walls they are directed upwards, providing the force to lift corrugated metal sheets from the rafters [28]. Torn corrugated metal is likely to cause significant harm to occupants compared to the softer thatching materials of the traditional *fale* [27]. Returning to indigenous architecture, traditional building practices, and using locally sourced materials may be essential in the development of disaster-resilient communities [6,7,27]. The Tongan *fale* is designed to allow high-velocity wind to pass through the open oval-shaped structure [9]. The curved contour of the roof means that wind moves around the surface without encountering resistance [9]. The building is secured with plaited rope of dried coconut fibre, which adds strength and provides greater flexibility [7]. If the traditional *fale* is damaged it can quickly be repaired, as construction materials are locally sourced and communities have the building skills required to make such repairs [7].

Another significant finding was the presence of mould in over a third of households. Mould thrives in damp, humid environments [16]. The major causes of dampness identified in the Tongan housing stock were evidence of water damage, ceiling leaks, and water ponding. Studies examining problems with micro-fungal infestation in damp buildings have determined that rising moisture from water leakage is one of the main determinants of household dampness [29]. While dampness in the home is not necessarily a cause of ill health in itself, it is a major determinant of several problematic exposures. This not only includes mould, but standing water can also lead to cockroach and rodent infestation [30]. A comprehensive removal of dampness sources and visible mould has been found to cause dramatic reductions in asthma exacerbations and respiratory illness [31]. Tongan families living in water-damaged homes would benefit from housing repairs to reduce water infiltration, and the removal of damaged building materials and repairs to windows to ensure adequate ventilation. Houses built on struts that are physically separated from the ground have more protection from groundwater, and should be considered in the face of sea-level rises and increased annual rainfall [5].

The Kids’Cam images also revealed possible sources of fire ignition, which could increase the risk of residential fires. These sources included open cooking fires, gas stoves, exposed electrical plugs and wiring, and the burning of rubbish and candles. These findings are consistent with that of the Tonga Fire Services, and contribute to the 57–68 structural fires that occur each year (2011 and 2012 data) [32]. The estimated loss associated with the reported structural fires is U.S. $1.2 million (2012) and results in injuries and deaths [32]. Simple interventions around the home could make a significant difference to reducing the incidence of fire. The risk of electrical fire ignition could be reduced by unplugging devices that are not in use from multi-plug adaptors and ensuring that electrical wiring is not exposed to water. In the cooking areas, gas cylinders could be safely stored outside and the gas hose run to the cooker through the external wall. Cooking areas and bedrooms should be fitted with functional smoke detectors, as this has been shown to reduce fatal fire injury by as much as 70% [33]. In some households, cooking areas are physically separate from the main house. This is a traditional layout that protects the house from a cooking fire by confining it to the cooking area [34]. The structure of the traditional *fale* is also built to minimise the spread and damage caused by fire, as it is constructed without the walls and pou (poles) used to support the roof [34].

Many households use bottled gas as their main source of energy for cooking, but solid fuel use is still commonplace and even preferred in some households that have gas stoves. Inside the cooking sheds, wood and coconut were used as fuel sources for open fires. These fuels produce indoor air pollution from the combustion byproducts of carbon monoxide, nitrous oxide, formaldehyde, and other poisonous gases [35,36]. Such gases cause respiratory illness, airway infections, coughs, wheezes, and asthma in susceptible populations [36]. Interventions to improve cooking practices, such as reducing the use of wood and charcoal, have been found to reduce the incidence of respiratory infections in households [35]. In line with the census data, much of the lighting in the houses was provided by fluorescent lighting [21]. Fluorescent light bulbs contain toxic chemicals such as mercury, and exposure to mercury from broken bulbs can cause mercury poisoning and acrodynia [37]. Fluorescent bulbs also emit light in a very wide band of wavelengths compared to residential light-emitting diode (LED) lights, which use 35–75% less energy and last up to 25 times longer [38]. Census data indicates that approximately 10% of household expenditure is spent on housing and utility costs in Tonga, so switching to energy efficient lighting and more efficient fuel sources for cooking may help reduce expenditure [21].

To our knowledge, this is the first study to objectively assess houses from a child’s perspective using wearable cameras automatically recording images at regular, frequent intervals. The wearable cameras enabled unprecedented access to the houses in which Tongan children live, and thus enabled an objective assessment of health risks and behaviours pertinent to housing. This methodology supplements previous methods of housing assessment, such as surveys, as it documents the children’s perspective of their house and facilitates an understanding of their living environment. Multiple risk factors and behaviours could be studied, and previously undescribed factors in the Tongan context can be identified, such as electrical risks and mould. It also overcomes the bias of self-report and the invasiveness of third-party observation. The method was practical, ethical, and acceptable to the children, their families, and the wider Tongan community. The collaborative research approach enabled the research to be undertaken in accordance with the needs and wishes of the Tongan community. It ensured that the analysis was appropriate and that the findings were disseminated.

The main drawback of this methodology is that the reliability of the coding decreases as the depth of interpretation increases. For example, the quantification of housing elements that contribute to building quality were at the discretion of the trained assessors and open to potential subjective biases. Moreover, the ability to consider the entire house is dependent on whether the images show all aspects of the house. In some cases, there were not enough images or the images’ content was insufficiently distinguishable to quantify certain aspects of the house.

While this is a small study, Tonga is sparsely populated with large families inhabiting each house. Further, participants were randomly selected and the consent rate was 100% with no drop-out. However, due to the sampling frame, only houses on the main island are included, and the number of urban houses in the study is higher than the proportion of the housing stock. Therefore, the generalisability of these findings should be viewed with caution, especially if there are substantial differences between housing on Tongatapu and the outer islands and in the quality of urban and rural houses. However, it is likely that the issues raised are still considerable.

The health risk factors and behaviours identified in this study can be modified with traditional practices and sustainable, community-led interventions. To improve the structural integrity of the housing stock, it is recommended that a bottom-up approach is taken by training and funding community leaders, tradespeople, and community members to be able to make improvements to strengthen existing housing. The benefits of traditional building materials and architecture should be promoted [27].

## 5. Conclusions

Western-style buildings constructed from cinder block, corrugated metal, and timber make up the Tongan housing stock. The majority of children in the study are living in houses that have evidence of multiple structural deficits and hazards that put them at an increased risk of injury, burns, and respiratory illness. Evidence of water damage was seen in almost one half of all houses, and mould was present in one third. Strengthening and repairing existing housing would reduce the associated health burden and increase the resilience of the housing to natural hazards. Community leaders, tradespeople, and community members can be trained to implement traditional building practices and provide practical solutions to commonly identified hazards, such as indoor gas cylinders, unsafe electrical arrangements, and house fire risk factors. To ensure that Tongan children live in a healthy and safe home, a collaborative approach between communities, community leaders, government, and NGOs is urgently needed. This research methodology and the findings from it may be of value to other Pacific Island nations and other developing countries.

## Figures and Tables

**Table 1:** The structural quality of the 72 households was assessed to determine the extent of damage and weather tightness to provide shelter and security for the occupants.

Structural Quality	Number of Households (*n*)	% of Total Households
Key structural indicators ^1^		
Evidence of interior water damage	30	42
Areas of visible mould	26	36
External walls/exterior cladding with extensive damage	24	33
Visible unrepaired holes in the roof or ceiling	18	25
Visible unrepaired holes in the internal walls	17	24
Other indicators		
Windows that are damaged or faulty	23	32
Insufficient number of windows to provide adequate lighting	8	11
Separate cooking shed in poor structural condition	7	10
Sleeping area not protected from outside environment	6	8

^1^ Eleven households had ≥ 4 of the five key structural observations and a total of nineteen households had ≥ 3.

**Table 2:** Risk factors and behaviours associated with negative health outcomes to which the children were exposed.

Risk Factors and Behaviours	Number of Households (*n*)	% of Total Households
**Electrical Hazards**		
Fluorescent lighting	64	89
Hanging electrical cords or extensions	51	71
Exposed, damaged, or dangerous electrical equipment or arrangements	33	46
**Cooking and Associated Risk Factors**		
Burn hazard from unsafe cooking practices or open flames	20	28
Inadequate surface space designated for food preparation	16	22
Gas cylinder(s) inside home	15	21
Knives or other cutting utensils left on the ground or open surface	11	15
Unhygienic environment for preparation or consumption of food	10	14
Barbeque / informal gas cooker inside home	5	7
Open fire inside home environment (solid fuels)	3	4
**Other Housing Hazards**		
Hazardous metal or exposed nails	16	22
Evidence of poor waste disposal facilities	15	21
Drying laundry inside house	10	14
Heavy objects with risk of falling and causing injury	10	14
Non-electrical sources of lighting	4	6
Broken or exposed glass	3	4

**Table 3:** Essential amenities and functions of the house.

Amenities and Functions	Number of Households (*n*)	% of Total Households
**Cooking Amenities**		
Refrigeration or freezer	55	76
Water collection tank on property	40	56
Gas cooker or stove	39	54
Sink in cooking area	37	51
Storage units in cooking area	32	44
Cooking on outside barbeque or open fire	9	13
Electrical cooker or stove	9	13
Separate cooking area to house	9	13
Vegetable garden	6	8
**Hygiene Related Amenities**		
Water collection tank on property	40	56
Running water inside house	38	53
Running water outside house	36	50
Washing machine for clothing	20	28
Hand washing of clothing	10	14
Flushing toilet	1	1
**Sleeping Amenities**		
Traditional sleeping arrangements	29	40
Bedroom(s) for child / children	26	36
Bedroom(s) for adult(s)	22	31
Mosquito net(s)	3	4
**Other Protective Factors**		
House surrounded by fencing	15	21
House on struts	5	7
Smoke alarm(s)	0	0

## Supplementary Materials

The following are available online at www.mdpi.com/1660-4601/14/10/1170/s1. Table S1: Coding schedule for the Kids’Cam Tonga housing project. Figure S1: Risk factors and behaviours observed in the home environment that impact on the risk of injury or harm to children: (**a**) Gas cylinder kept outside of the cooking area by running the gas hose through external wall (protective); (**b**) Preparing an open fire with solid fuel sources (coconut, wood and coal) and using petrol to ignite the fire. Fire is in close proximity to the house; (**c**) Rubbish and waste dumped at the back of property; (**d**) Pig sty surrounding an area that is used for food preparation and eating. Figure S2: Structural damage and building quality of housing stock: (**a**) House constructed from metal sheet and patched up with lumber, cardboard and fabric; (**b**) House with damaged window structures and insufficient natural lighting inside; (**c**) Evidence of rotten wood and water damage to door frame; (**d**) Ceiling with evidence of water damage and ceiling tiles missing or replaced with plastic sheets.
