# Supplementary material for: Housing as a Determinant of Tongan Children’s Health: Innovative Methodology Using Wearable Cameras
Source: Int J Environ Res Public Health. 2017 Oct 4;14(10):1170. doi: 10.3390/ijerph14101170 (PMC5664671; doi:10.3390/ijerph14101170)
Supplement: Supplementary file 1 [file ijerph-14-01170-s001.pdf]

International Journal of
Environmental Research and Public Health

MDPI

Supplementary Figures

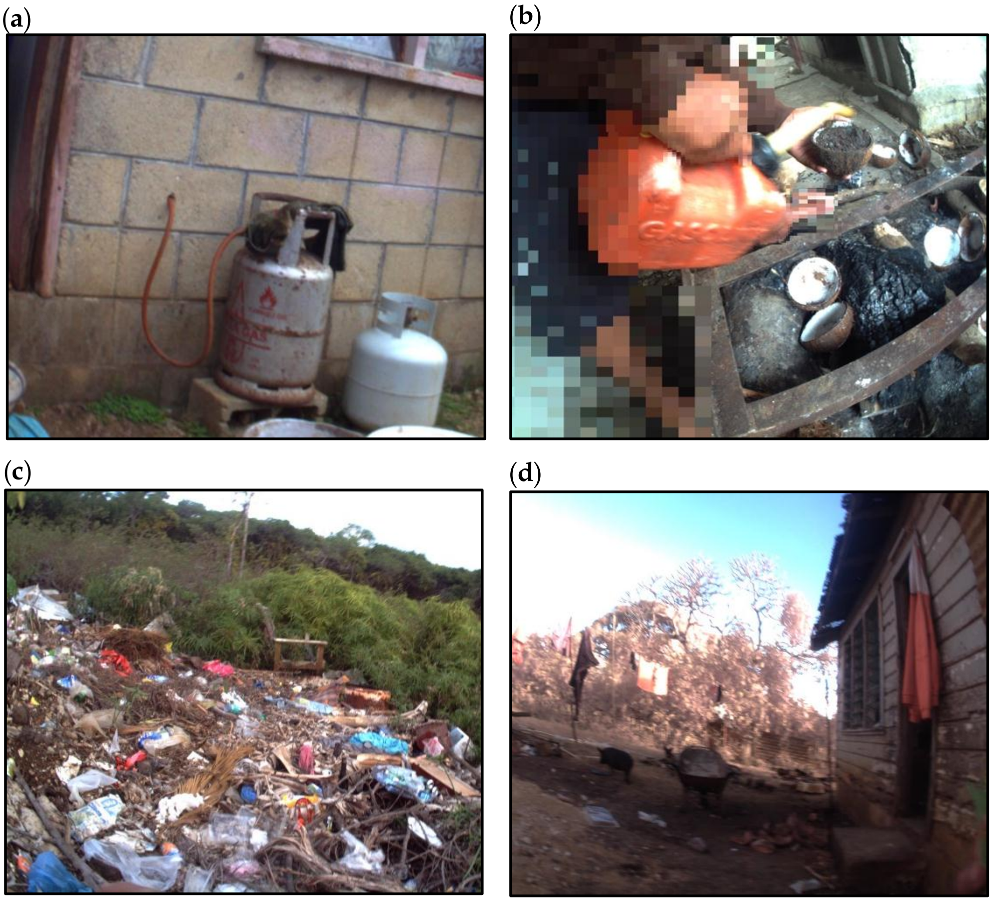

Figure S1. Risk factors and behaviours observed in the home environment that impact on the risk of injury or harm to children: (**a**) Gas cylinder kept outside of the cooking area by running the gas hose through external wall (protective); (**b**) Preparing an open fire with solid fuel sources (coconut, wood and coal) and using petrol to ignite the fire. Fire is in close proximity to the house; (**c**) Rubbish and waste dumped at the back of property; (**d**) Pig sty surrounding an area that is used for food preparation and eating.

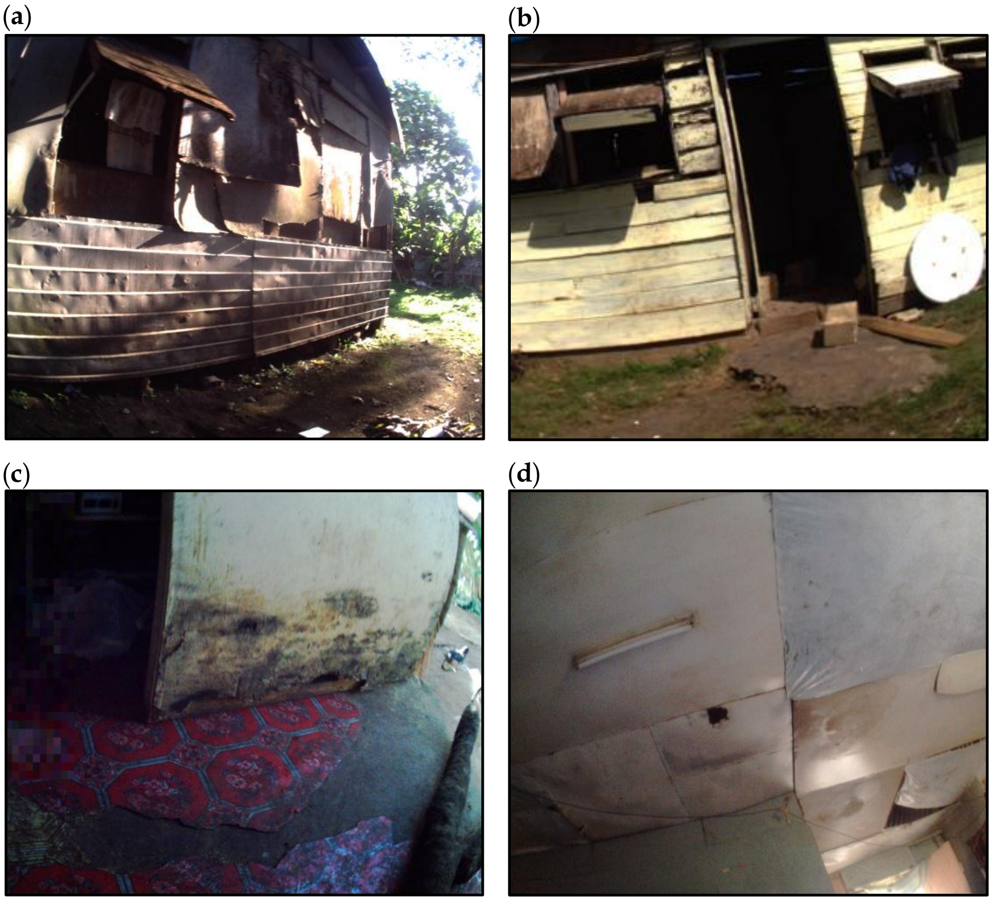

Figure S2. Structural damage and building quality of housing stock: (**a**) House constructed from metal sheet and patched up with lumber, cardboard and fabric; (**b**) House with damaged window structures and insufficient natural lighting inside; (**c**) Evidence of rotten wood and water damage to door frame; (**d**) Ceiling with evidence of water damage and ceiling tiles missing or replaced with plastic sheets.

Supplementary Table S1. Coding schedule for housing building materials, structural quality, housing risk factors and behaviours, and amenities and housing function.

Building Materials	Description
Cinder block or concrete exterior	Primary building material used in exterior of house is cinder block, besser block or similar concrete masonry. The house has a Western-style design.
Corrugated metal roof	Primary building material present in roof structure is corrugated aluminium or similar sheet metal.
Corrugated metal sheet exterior	Primary building material used in exterior of house corrugated aluminium or similar sheet metal. The house has a Western-style design.
Timber based roof	Primary building material present in roof structure is timber, chipboard or other processed wood.
Timber exterior	Primary building material used in exterior of house is timber, chipboard or other processed wood. The house has a Western-style design.
Traditional Tongan *fale*	Domed-shaped roof thatched with coconut leaves, sugar cane or other raw materials. Roof sitting on timber supports and bound together with coconut fibre or ropes.

Structural Quality	Description
Areas of visible mould	Extensive covering of a surface in clusters of black /grey spots which could not reasonably be dirt or soot and associated with a source of moisture or direct water contact.
Evidence of interior water damage	Evidence of at least one of: water stains on the ceiling, rotten wood / timber, cracked or peeling paint on ceiling, plastic or other material being using to cover ceiling hole to which water could enter.
External walls / exterior cladding with extensive damage	Evidence of damage to the primary building materials and/or masonry covering the exterior of the house. Including, but not limited too, rusted aluminium sheets, incomplete or inappropriate repairs, loss of cladding and split timber.
Insufficient number of windows to provide adequate lighting	House appears dark during daylight hours and rooms have no or very few windows which provide natural light.
Separate cooking shed in poor structural condition	Deliberate area designated for the preparation or cooking of food and distinctly separated from the house structure. Poor condition defined as appearance of mould, rubbish, unhygienic practices and significant structural damage.
Sleeping area not protected from outside environment	The area in which the beds (mattresses or traditional arrangement) are organised has structural damage such that there are holes in the ceiling and walls, or other structural evidence that suggests the occupant would not be protected from environmental factors (rain, extreme temperature, pests) at night.

Visible unrepaired holes in the internal walls	Holes that can be clearly identified in the internal walls from an interior view. Natural light can be seen through the hole indicating a direct breach of the internal and external wall.
Visible unrepaired holes in the roof or ceiling	Holes that can be clearly identified in the roof or ceiling from an interior view. Natural light can be seen through the hole indicating a direct breach of the internal ceiling and external roofing.
Windows that are damaged or faulty	Windows that are boarded shut so that they are not able to be opened or closed. Windows that are broken leaving large gaps in the house structure.

**Risk factors and Behaviours**	**Description**
Barbeque / informal gas cooker inside home	House contains a barbeque / informal gas cooker identified by portable appearance and unprotected flame as well as gas cylinder clearly visible. See definition of gas cylinder.
Broken or exposed glass	Broken or shattered glass from windows, mirrors, bottles or other sources of glass inside the house or surrounding area.
Burn hazard from unsafe cooking practices or open flames	Open fire or stove that is unsupervised and in close proximity to young children present. Hazardous cooking arrangement involving the ignition, fuel source or exposure to the flame. Also includes scald hazards from boiling water or hot metal placed on unstable surface.
Drying laundry inside house	Clothing or fabrics hanging from lines within one of the living areas of the house and appear to be damp or wet.
Evidence of poor waste disposal facilities	Large amounts of rubbish or waste that is persistently on the house property accompanied by lack of rubbish bins or recycling facilities.
Exposed, damaged or dangerous electrical equipment / arrangements	Evidence of at least one of: electrical equipment that is exposed to water or rain, overloading of electrical extension blocks, rusting plugs or electrical components, exposed wiring or power cords.
Fluorescent lighting	Use of fluorescent bulb(s) for lighting. Identified at night by appearance of extended bar of fluorescent light. Visualized in daylight as long glass fluorescent tube usually in ceiling fixture or high up on wall. Predominant source of lighting in house.
Gas cylinder(s) inside home	Presence of any size of gas cylinder(s), such as metal LPG gas bottles, inside the house or within enclosed cooking areas. Gas bottles identifiable by hazard symbol, orange hose and proximity to gas appliance.
Hanging electrical cords or extensions	Electrical cords hanging down from walls, ceiling, windows and are present at a height that could reasonably cause a trip or other injury.
Hazardous metal or exposed nails	Any of: scrap metal, metal with sharp or rusting edges or exposed nails in areas where occupants walk and on the ground or at a height that could potentially cause injury.
Heavy objects with risk of falling and causing injury	Heavy items at a height and location that they could reasonably cause an injury should the object fall on top of a child.

Inadequate surface space designated for food preparation	No deliberate space allocated for the preparation of food. Chopping food without supporting surface, preparing food on the ground or preparing raw meats on surfaces used for other non-food related activities.
Knives or other cutting utensils left on the ground or open surface	Knives or sharp cutting utensils left in areas that are easily accessible to the young children present or on the ground and could be stepped on.
Non-electrical sources of lighting	Candles, paraffin lamps, battery torches or other non-electrical fuel sources used for lighting.
Open fire inside home environment (solid fuels)	Open fire inside the house or enclosed cooking areas. Open fire defined by exposed, unprotected flame burning solid fuel sources such as wood, coal, coconut or waste.
Unhygienic environment for preparation or consumption of food	Preparation or consumption of food on surfaces that are covered in dirt or mould or soot. Animals (pigs and dogs) interacting with the surface used to prepare or consume food.

**Amenities and Functions**	**Description**
Bedroom(s) for adult(s)	Defined, permanent space(s) allocated to adult(s) for sleeping. Indicated by presence of adult(s) sleeping on mattress and bed(s) in a single room.
Bedroom(s) for child / children	Defined, permanent space(s) allocated to child / children for sleeping. Indicated by presence of child / children sleeping on mattress and bed(s) in a single room.
Cooking on outside barbeque or open fire	Open fire or outside barbeque present on property or semi covered veranda. Barbeque defined as a portable, gas system with a platform for cooking food. Open fire identified by the burning of solid fuel sources (wood, coal, coconut) and the utilisation of the flame for cooking.
Electrical cooker or stove	Cooker or stove powered by electricity. Identified by evidence of connection to electrical supply, absence of flame when cooking on elements, or model and brand.
Flushing toilet	Flushing toilet inside the house or in a separate external building on the property.
Gas cooker or stove	Cooker or stove identified by gas flame around element, presence of gas cylinder or characteristic components of cylinder (orange hose) or recognisable model and brand.
Hand washing of clothing	Clothing being washed in basins or buckets by hand and no evidence of machine use.
House on struts	House physically elevated from the ground by struts or blocks to allow water to pass below.
House surrounded by fencing	Clear fence, wall or border that outlines the property and provides some degree of separation from the surrounding area.

Mosquito net(s)	Mesh of fabric mounted or draped over a sleeping area to provide protection from mosquitoes, flies and other insects.
Refrigeration or freezer	Refrigeration or freezer identifiable by electrical connection, food storage visible when open and common brands and models.
Running water inside house	Potable drinking water supplied by a tap inside the house or cooking area.
Running water outside house	Potable drinking water supplied by a tap outside of the house.
Separate cooking area to house	Area allocated specifically for the preparation and cooking of food. Structurally detached from the main house.
Sink in cooking area	Permanent structure situated underneath internal water source to collect water. May be used for washing dishes, preparing food and general hygiene.
Smoke alarm(s)	Smoke detector(s) present on the ceiling or wall of any room and in normal functional appearance.
Storage units in cooking area	Deliberate cupboards and shelving space allocated specifically for the storage of food, utensils and cooking supplies. Located in food preparation and cooking areas.
Traditional sleeping arrangements	Children and/or adults sleeping in a temporary arrangement together. Indicated by individuals sleeping on mats, sofas or removable mattress in a single living area.
Vegetable garden	Deliberate area of property allocated for the growth of vegetables and plants. Evidence of maintained soil, appearance of vegetables and active gardening confirm presence.
Washing machine for clothing	Washing machine identifiable by connection to electrical source, loading or removal of clothing from machine and appearance of common models / brands.
Water collection tank on property	Water tank container for storing rain water collected by roofing system. Usually concrete structure.
